# Total Hip Arthroplasty for Hip Fracture: Clinical Results and Mid-Term Survivorship

**DOI:** 10.7759/cureus.20492

**Published:** 2021-12-17

**Authors:** James R Gill, Aly Pathan, Samuel J Parsons, Konrad Wronka

**Affiliations:** 1 Trauma and Orthopaedics, West Suffolk Hospital NHS Foundation Trust, Bury St Edmunds, GBR

**Keywords:** fragility fracture, neck of femur fracture, total hip arthroplasty, intracapsular, hip fracture

## Abstract

Aim

The purpose of this study is to report the clinical results and mid-term survivorship for total hip arthroplasty (THA) performed to treat displaced intracapsular hip fracture.

Methods

Between January 2005 and December 2019, 414 patients underwent THA for acute displaced intracapsular hip fracture. The mean age of patients was 73.2±8.0. Out of the total patients, 89.6% received a cemented THA. Kaplan-Meier analysis was performed with implant survivorship and dislocation as separate endpoints. Complications and modified Harris Hip Score (HHS) at the latest follow-up were also reported.

Results

There was a total of nine revisions, six were performed for dislocation and three for infection. Kaplan-Meier implant survivorship was 99.0% at five years and 97.7% at ten years. Twenty (4.8%) patients suffered at least one dislocation and 11 suffered more than one, so following a first dislocation, the chance of one or more further dislocations was 11/20 (55%). Kaplan-Meier analysis with dislocation as the endpoint showed the risk of dislocation of 2.9, 4.4, and 5.2% at one, five, and ten years respectively. Three (0.7%) patients suffered late periprosthetic fractures and 22 (5.3%) contralateral hip fractures. The mean modified HHS at the latest follow-up was 86.3±18.9.

Conclusion

In conclusion, the present study shows excellent implant survivorship, good hip function, and moderate risk of dislocation and contralateral hip fracture following THA for displaced intracapsular hip fracture. Our study shows favourable results for THA treatment in a selected group of patients with hip fractures.

## Introduction

The annual incidence of hip fracture in the United Kingdom (UK) is approximately 77 000 [1]. In elderly patients approximately half of the hip fractures are intracapsular and two-thirds of these are displaced [2]. In younger patients, intracapsular fractures are usually treated with reduction and internal fixation because the priority is joint preservation. There is a relatively high risk of non-union and osteonecrosis associated with the fixation of displaced intracapsular fractures. In elderly patients, the standard of practice is arthroplasty because the priority is early full weight-bearing and avoidance of complications requiring further surgical intervention [3].

The two options for arthroplasty for intracapsular hip fracture are either hemiarthroplasty or total hip arthroplasty (THA). Hemiarthroplasty is the procedure of choice for elderly patients with low physical demands as the procedure provides satisfactory mobility and pain relief with a relatively low risk of reoperation. Studies have shown that in more mobile and active patients hemiarthroplasty may not allow patients to mobilise to their full potential [4], and in the long-term result in acetabular erosion [5]. Since 2011 the National Institute for Health and Care Excellence (NICE) has recommended offering THA for displaced intracapsular hip fracture for patients who walk with no more than one stick, who are not cognitively impaired, and are fit to undergo anaesthesia and the procedure [6]. The perceived advantages of THA for hip fracture are better mobility, less pain, and reduced risk of acetabular erosion [4]. The disadvantages of THA are longer anaesthetic, increased intraoperative blood loss, increased risk of dislocation and reoperation, increased cost compared to hemiarthroplasty, and difficulties with service provision [7].

THA versus hemiarthroplasty for the treatment of the displaced intracapsular hip fracture is an area of continuing contention in the orthopaedic literature. A systematic review and meta-analysis reported THA to be superior to hemiarthroplasty in terms of risk of reoperation, hip function, and quality of life [4]. However, some recent studies have questioned the benefit of THA compared to hemiarthroplasty [8-11]. There is a paucity of published data reporting the long-term outcomes of THA for hip fracture. The aim of this study is to report the clinical and survivorship results of THA performed to treat displaced intracapsular hip fracture at our institution between 1st January 2005 to 31st December 2019.

## Materials and methods

Institutional review board approval was granted for this retrospective case series (West Suffolk Hospital Research and Development Approval Number: 2020SERV005). A search of our hospital operations database was conducted to identify all THA procedures performed for acute traumatic hip fracture between January 2005 and December 2019. Inclusion criteria were all intracapsular fractures treated with THA. Exclusions were patients treated with THA for failed internal fixation of hip fracture, a proximal femoral replacement for hip fracture, and hip fracture with osteonecrosis or severe osteoarthritis precluding hemiarthroplasty.

The electronic patient records and imaging were reviewed in order to determine the characteristics of all patients, procedures, and complications. Our institution conducts a joint surveillance programme for all patients who undergo any total joint arthroplasty procedure. All patients who have undergone THA including those who have suffered a femoral neck fracture have plain film radiographs at one, five, and ten years postoperatively and are invited to complete a modified Harris Hip Score (HHS) one and five years following the index procedure. The modified HHS includes an assessment of pain (44 points) and function (47 points). The modified HHS lacks assessment for the deformity (4 points) and range of motion (5 points) which require a physical examination and result in the original HHS being measured out of 100. Using a multiplier of 1.1 (100/91) applied to the modified HHS provides a total possible score of 100 and allows comparison with the original HHS. The modified HHS has been used in previous studies to assess outcomes following fractured neck of the femur [4,10,12].

Between January 2005 and December 2019, we identified 2533 patients who were treated with hemiarthroplasty or THA for an acute displaced intracapsular neck of femur fracture. 414 (16%) were treated with THA and 2119 (84%) underwent hemiarthroplasty. A total of 15 different surgeons performed or supervised THA in this series. In 76 (18%) of the procedures, the primary surgeon was a speciality registrar supervised by a consultant surgeon. Most but not all of the surgeons were lower limb arthroplasty specialists. Thirteen of the surgeons used a posterior surgical approach and two used an anterior-lateral approach. Patient, implant, and surgical characteristics are displayed in Tables 1, 2.

**Table 1:** Characteristics of patients receiving THA treatment THA = Total hip arthroplasty; BMI=Body Mass Index; N = Total number of patients; n = number of patients

Characteristics	N=414
Age (years), mean±SD (range)	73.2±8.0 (49.2-96.7)
Gender, n female (%)	308 (74.4)
Laterality, n right (%)	220 (53.1)
BMI (kg/m^2^), mean±SD (range)	25.1±4.5 (15.3-46.1)

**Table 2:** Implant and surgical characteristics of patients receiving THA treatment THA = Total hip arthroplasty; n = Number of patients

Characteristics	Implant	Implant details	n
Classification of THA, n (%)	Cemented		371 (89.6)
	Hybrid		40 (9.7)
	Uncemented		2 (0.5)
	Reverse hybrid		1 (0.2)
Femoral stem, n (%)	Cemented	Exeter V40® (Stryker, Newbury, UK)	411 (99.3)
	Uncemented	POLARSTEM (Smith & Nephew, Baar, Switzerland)	3 (0.7)
Acetabular cup, n (%)	Cemented	Marathon (DePuy International Limited, Leeds, UK)	159 (38.4)
		Ogee Elite (DePuy International Limited, Leeds, UK)	129 (31.2)
		Exeter Contemporary (Stryker, Newbury, UK)	57 (13.8)
		REFLECTION (Smith & Nephew, Baar, Switzerland)	12 (2.9)
		Cenator (Corin Group PLC, Cirencester, UK)	15 (3.6)
	Uncemented	R3 (Smith & Nephew, Baar, Switzerland)	41 (9.9)
		Trident (Stryker, Newbury, UK)	1 (0.2)
Head size (mm), n (%)		22.5	4 (1)
		26	113 (27.3)
		28	289 (69.8)
		32	6 (1.4)
		36	2 (0.5)
Surgical approach		Posterior	358 (86.5)
		Anterior-lateral	56 (13.5)

The primary outcome of this study was implant survivorship. Secondary outcomes were dislocation, mortality, periprosthetic THA fracture, contralateral hip fracture, and modified HHS. Individual surgeon data was checked with the National Joint Registry (NJR) to confirm no cases of revision were overlooked. The rate of revision performed for dislocation was also reported per 1000 patient-years follow-up to allow comparison with NJR data.

Statistical analysis

Data collection and descriptive statistics were performed with Excel version 16 (Microsoft, Redmond, USA). Fisher's exact test was used to compare categorical data and a p-value <0.05 was considered statistically significant. Separate Kaplan-Meier survivorship analyses were performed with joint revision, dislocations, and patient mortality as the endpoints. A revision was defined as an operation in which any new components were added or at least one of the components or the bearing surface was changed.

## Results

Revisions

Nine THAs were revised at a mean time of 26.6 months from primary THA. Details of revisions are displayed in Table 3.

**Table 3:** Details of revision arthroplasty PLAD = Posterior lip augmentation device DAIR = Debridement, antibiotics and implant retention *performed at a tertiary referral centre

Age	Sex	Time (months)	Reason for revision	Revision procedure
78	F	0	Dislocation	PLAD
73	F	25	Dislocation	PLAD
72	F	64	Dislocation	PLAD
72	M	80	Dislocation	PLAD
84	F	1	Dislocation	Revision of cemented stem
79	F	10	Dislocation	Revision to a bipolar head and constrained cup
77	F	62	Infection	Two-stage revision*
62	F	1	Infection	DAIR (stem and head revised)
67	F	8	Infection	DAIR (head revised)

**Figure 1:**
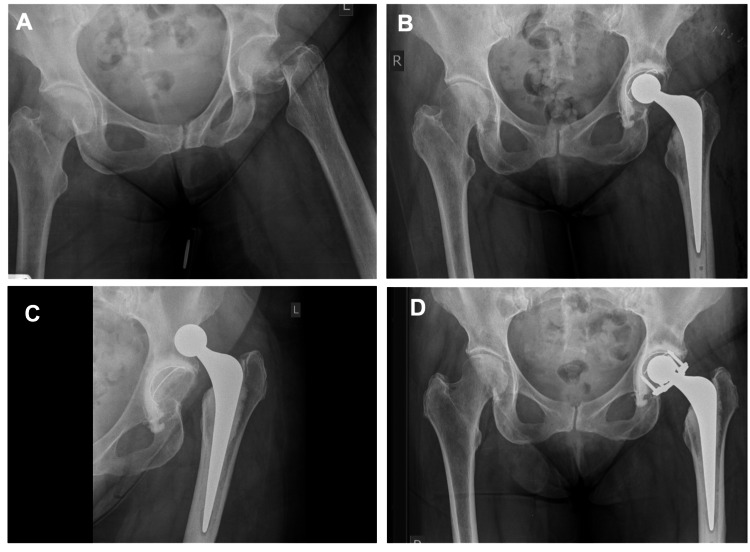
Plain film anteroposterior radiographs in the case of a 72-year-old female A. Left intracapsular fractured neck of femur; B. Cemented total hip arthroplasty (THA); C. Posterior-superior dislocation; D. Reduced THA with posterior lip augmentation device

Dislocation was identified as the most common reason for revision. Of the four patients who were treated with a posterior lip augmentation device (PLAD) (DePuy International Limited, Leeds, UK) three suffered no further dislocations (Figure 1). The remaining patient treated with a PLAD (five years post-THA) suffered a fall and sustained a periprosthetic fracture one year following PLAD which was re-revised to a long uncemented distally locked stem Arcos® (Zimmer Biomet, Warsaw, USA). This was complicated by a further dislocation six months following re-revision. One stem was revised for dislocation with a cement-in-cement revision using a 125mm Exeter V40® stem (Stryker, Newbury, UK) and subsequently suffered one further dislocation two weeks later which was successfully treated with an abduction brace.

**Figure 2:**
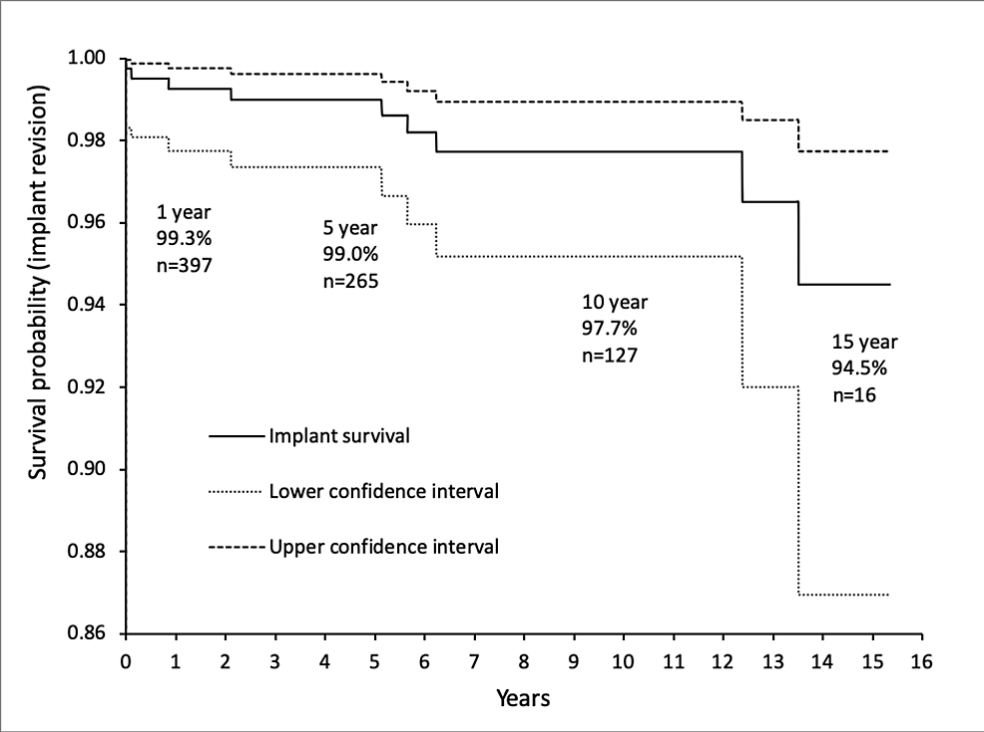
Kaplan-Meier survivorship analysis of total hip arthroplasties performed for fractured neck of femur with revision as the endpoint

The remaining revision for dislocation was treated with revision of a cemented cup to a bipolar constrained cemented acetabular cup Trident® (Stryker, Newbury, UK). Three revisions were performed for infection. One underwent a two-stage revision at a tertiary referral unit and two underwent successful debridement, antibiotics, and implant retention (DAIR) at our institution. None of the cases of revision for infection were complicated by dislocation. No cases were revised for aseptic loosening. Including the DAIR and PLAD procedures, a total of nine cases were included in the implant survival analysis. Kaplan-Meier implant survivorship was 99.0% and 97.7% (95% confidence intervals 99.6 to 97.4 and 98.9 to 95.2) at five and ten years respectively (Figure 2). 

Dislocation

**Figure 3:**
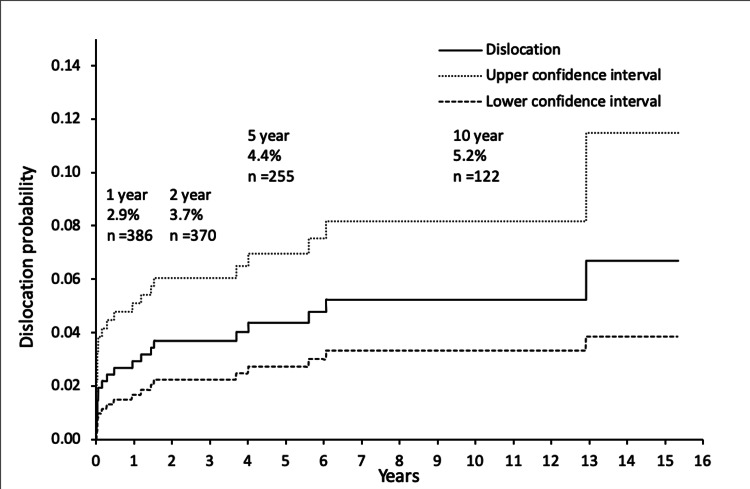
Kaplan-Meier analysis of total hip arthroplasties performed for fractured neck of femur with dislocation as the endpoint

Twenty patients suffered a dislocation (4.8%) at a mean time to the first dislocation of 14 months. Eleven patients suffered more than one dislocation, so following a first dislocation, the chance of suffering one or more further dislocations was 11/20 (55%). The total number of dislocations suffered by these twenty patients was 49. Kaplan-Meier analysis with dislocation as the endpoint showed a risk of dislocation of 2.9, 3.7, 4.4, and 5.2% at one, two, five, and ten years respectively (Figure 3). A total of six revisions were performed for dislocation. The mean follow-up of patients in this series was 5.35 years, taking mortality into account. Therefore, 2.7 revisions for dislocation were performed per 1000 patient-years in our series. The head size was 26mm for eight patients and 28mm for 12 patients who suffered a dislocation. Dislocation occurred in two cases performed via an anterior-lateral approach and 18 cases performed via a posterior approach. There was no statistical difference in the rate of dislocation according to head size (p=0.3056) or approach (p=1.0000).

Mortality

**Figure 4:**
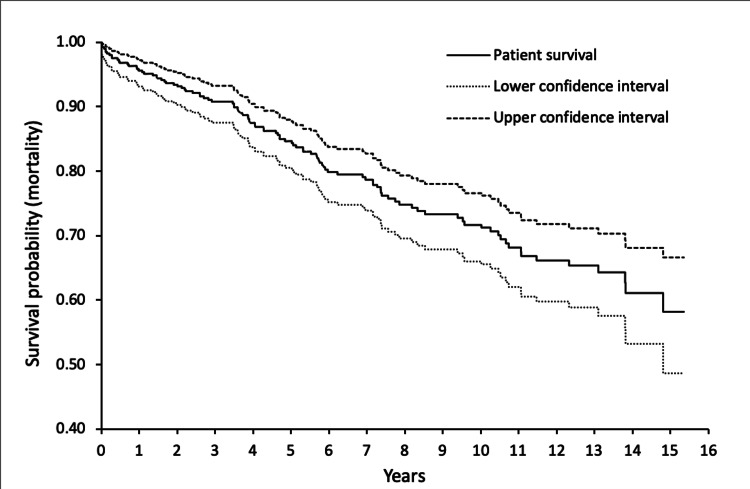
Kaplan-Meier Survivorship analysis of total hip arthroplasties performed for fractured neck of femur with patient mortality as the endpoint

Kaplan-Meier survivorship analysis showed that patient survivorship was 99.0% at 30 days, 98.1% at three months, 95.6% at one year, 84.7% at five years, and 71.2% at 10 years (Figure 4).

Periprosthetic fracture

Three patients (0.7%) suffered periprosthetic fractures at 9, 80, and 92 months after THA. All three cases involved cemented stems. Two were treated with open reduction and internal fixation with a plate (Vancouver B1 [well-fixed stem] and B2 [loose stem with good bone stock]) and one was treated with revision to a long uncemented stem with cable fixation (Vancouver B2). This was a re-revision as the patient had already undergone revision with PLAD for a late dislocation.

Contralateral hip fracture

Twenty-two patients (5.3%) suffered a contralateral hip fracture at a mean time of 47 months after THA. Thirteen patients suffered displaced intracapsular fractures, two were treated with THA, and 11 were treated with hemiarthroplasty. The remaining nine patients suffered extracapsular fractures which in all cases were treated with a dynamic hip screw.

Modified Harris Hip Score

The modified HHS was available for 280 patients out of 361 (78%) patients who were alive and had completed one year of follow-up. The mean modified HHS at the latest follow-up was 86.3±18.9. The latest follow-up was five years post THA for 75 patients and one year for 205 patients resulting in a mean follow interval of 25 months for the modified HHS. The mean modified HHS was 87.9±17.1 and 80.6±19.5 at one- and five-year follow-up respectively.

## Discussion

The principal findings from this study are: excellent medium-term survivorship for THA following hip fracture, low incidence of late periprosthetic fracture, good patient-reported outcomes, and moderate risk of dislocation and contralateral hip fracture.

The implant survivorship results reported in this study are superior to those reported by joint registries for hip fracture [13]. The National Joint Registry (NJR) reported five- and ten-year implant survivorship of approximately 97.3 and 94.8% following a hip fracture [14]. The Swedish Hip Arthroplasty Register (SHAR) reports 93.3% implant survivorship of cemented Exeter THA for hip fracture at 12 years [15]. The excellent survivorship reported in this study is in part due to implants used, namely the Exeter V40® stem [16]. There is a paucity of long-term survivorship data reported in the literature for THA performed for hip fracture [4,10].

The most common reason for revision in the present study was dislocation. The NJR reports 0.86 revisions per 1000 patient-years for dislocation for cemented metal on polyethylene (MoP) THA, irrespective of indication, whereas the risk of revision for dislocation in the present study was 2.7 per 1000 patient-years [14]. Therefore, the risk of undergoing a revision for dislocation in the present study is 3.1 times greater compared to all primary MoP THAs recorded in the NJR. The increased risk of dislocation associated with THA for hip fracture has been reported extensively in the literature and is one of the primary concerns with offering THA. The risk of dislocation associated with THA for hip fracture is greater than that reported for both, hemiarthroplasty performed for hip fracture [17] and THA performed for osteoarthritis [14].

The rate of dislocation in this study is similar or less than published rates of dislocation for conventional THA for hip fracture [9,12,18]. In a meta-analysis comparing THA to hemiarthroplasty for hip fracture, Lewis et al. reported a dislocation rate of 8.1% in 591 THA compared to 2.7% in 630 hemiarthroplasties with varying durations of follow-up [4]. In a randomised controlled trial (RCT) comparing THA to hemiarthroplasty, the Hip Fracture Evaluation with Alternatives of Total Hip Arthroplasty versus Hemi-Arthroplasty (HEALTH) investigators reported dislocation rates of 4.7% for THA compared to 2.4% for hemiarthroplasty, two years post-hip fracture surgery [8]. In a study using data from the SHAR, Hansson reported a dislocation rate of 8% in over 6000 THAs performed for hip fracture [19].

The majority of femoral heads used in the present study were size 28mm or smaller. Using head sizes 32mm or larger has been shown to reduce the risk of dislocation in primary THA [20]. Coomber et al. published a review of the evi­dence on technique for performing THA for hip fracture and recommend the use of larger head sizes (32 mm to 36 mm) [21]. Dual mobility components have been shown to reduce the risk of dislocation further in patients with a femoral neck fracture [22].

There has been debate about which is the best surgical approach when performing THA for hip fracture [13]. Matharu et al. conducted an NJR study examining the effect of the surgical approach on outcomes following THA for hip fracture [23]. The authors showed that with a posterior approach survival rates were significantly higher and intraoperative complications significantly lower with no significant difference in risk of revision due to dislocation compared to an anterior-lateral approach.

A number of factors may contribute to the increased risk of dislocation following THA for hip fracture compared to elective primary THA for osteoarthritis (OA). In terms of patient factors, hip fracture patients are at increased risk of falls, have less pre-operative counselling and education. Hip fracture patients often go from having a normal hip to THA, whereas patients with OA have often endured a painful hip with a limited range of motion for months if not years before arthroplasty and have had to change their behaviours to compensate for this over a long period of time. In terms of surgical factors, there is the absence of a thickened hip joint capsule which is associated with OA, templating is trickier and the anatomy is distorted following the fracture, making recreation of native offset and leg length more challenging.

Mortality reported in the present study is favourable compared to the published literature [13]. The meta-analysis by Lewis et al. reported a mortality rate of 61/460 (13.3%) from eight studies with follow-up ranging from 2-13 years [4]. The HEALTH investigators reported a mortality rate of 14.3% two years post THA for hip fracture [8] and Viswanath et al. reported 10-year mortality of 33.3% in a single centre series of patients that underwent THA for hip fracture [9].

The rate of late periprosthetic fracture in the present study was 3/414 (0.7%) compared to a contralateral hip fracture rate of 22/414 (5.3%). So, patients were seven times more likely to suffer a contralateral hip fracture than periprosthetic fracture. The rate of contralateral hip fracture highlights the importance of fall prevention interventions and treatment of osteoporosis following a hip fracture [24]. In the RCT by the HEALTH investigators, the incidence of periprosthetic fracture within two years of hip fracture surgery was 5.3% for THA and 4.8% for hemiarthroplasty [8]. The rate of periprosthetic fracture reported in the present study is comparable to that reported for elective primary THA [18,25]. Meek et al. reported a periprosthetic fracture incidence of 0.9% five years post-primary elective THA in a registry study of over 50,000 THAs [26].

The clinical results reported in the present study are comparable to results reported in the literature for THA performed for hip fracture [4,10-12]. In an RCT comparing THA to hemiarthroplasty for hip fracture, Van Den Bekerom reported a modified HHS of 76.0 at one year and 75.2 at five-year follow-up [12]. Eighteen patients from the same study were available for 12-year follow-up at which time the modified HSS was 69.3 [10]. In a meta-analysis of THA versus hemiarthroplasty for hip fracture, Lewis et al. reported a mean HHS of 83.1 in 236 patients with varying durations of follow-up [4].

One of the greatest challenges for surgeons faced when treating elderly patients with displaced intracapsular neck of femur fractures is to determine which patients might benefit from a THA compared to a hemiarthroplasty [27]. NICE guidance merely recommends THA should be offered to patients with a displaced intracapsular fracture who are able to walk independently out of doors with no more than the use of a stick, not cognitively impaired, and medically fit for anaesthesia and the procedure. Nationally, the proportion of eligible patients that receive a THA from 2015, when reports began, to 2019 is 31.08%, whereas the average proportion at our institution during the same period has been 32.3%. So, the proportion of eligible patients that receive a THA for hip fracture at our institution reflects national practice. In a study, which analysed National Hip Fracture Database data, Perry et al. characterised which factors are important in deciding which patients receive a THA for hip fracture in UK practice [27]. Perry et al. showed that age is the most important factor, with age 76 representing a split in the decision tree [27]. In terms of mobility guiding decision for THA, surgeons were more restrictive than the recommendations by NICE, the most important partition in mobility was the difference between mobilising independently without aids and mobilising with aids.

The results reported in the present study are superior to the results reported by a number of RCTs [4,8,11]. Most recently the HEALTH investigators reported no difference in secondary procedures, similar mortality, and modestly but not significantly superior function following THA compared to hemiarthroplasty [8]. The HEALTH investigators recruited patients using criteria similar to the NICE guidelines, the mean age of patients recruited to the trial was 79 years [28]. Likewise, the HOPE-Trial which randomised patients greater than 80 years of age to THA or hemiarthroplasty following displaced intracapsular fracture found no difference in hip function or quality of life at two-year follow-up [11]. The mean ages of patients in both of these RCTs are greater than the present study as well as current UK practice [27]. The HEALTH investigators have shown that the benefits of THA over hemiarthroplasty are marginal in patients with a mean age of 79 years and using inclusion criteria similar to the NICE guidelines with two-year follow-up. However, this does not mean younger and more active patients would not benefit from THA as opposed to hemiarthroplasty. The present study shows that a certain group of elderly patients does well following intracapsular hip fracture treated with THA. Patient selection is critical in order to achieve successful clinical outcomes and the consequence of poor selection can be devastating. As patient activity levels decline not only do the potential functional benefits of THA decrease, but the systemic risks of surgery and local complications also increase. There is a tipping point whereby hemiarthroplasty is the more appropriate operation [29]. One of the most important and urgent areas for further research will be to determine where this tipping point lies and therefore evidenced-based indications for THA [7,30].

There are a number of limitations associated with this study. Firstly, this study was a retrospective review. Secondly, only one outcome score, the modified HHS was used to assess function and no patients were evaluated beyond five-year follow-up. Thirdly, although all revisions which took place in the UK should have been captured by the NJR this is not the case for dislocations and periprosthetic fractures. It is possible that patients presented to another institution for treatment of a dislocation or a periprosthetic fracture which did not result in revision. Fourthly, there were no strict selection criteria for eligibility for THA, the final decision for THA was made by the operating surgeon. And finally, data were not available to allow comparison to patients who received a hemiarthroplasty.

## Conclusions

In conclusion, the present study shows excellent implant survivorship, good hip function, and moderate risk of dislocation and contralateral hip fracture following THA for displaced intracapsular hip fracture. Our study shows that despite recent evidence questioning the benefits of THA for hip fracture in a selected group of patients results are favourable. Current patient selection guidelines are inadequate because of a paucity of evidence. Further research is required to determine evidence-based indications for the treatment of hip fracture with THA.
